# Evaluation of reproductive performances of the common octopus (*Octopus vulgaris*) reared in water recirculation systems and fed different diets

**DOI:** 10.1038/s41598-020-72151-y

**Published:** 2020-09-17

**Authors:** Antonio Casalini, Alessandra Roncarati, Pietro Emmanuele, Niccolò Guercilena, Alessio Bonaldo, Luca Parma, Oliviero Mordenti

**Affiliations:** 1 Department of Veterinary Medical Sciences – DIMEVET, University of Bologna, Via Tolara di Sopra 50, 40064 Ozzano dell’Emilia, BO Italy; 2 School of Biosciences and Veterinary Medicine, University of Camerino, Viale Circonvallazione 93/95, 62024 Matelica, MC Italy

**Keywords:** Biological techniques, Zoology

## Abstract

The reproductive performance of *Octopus vulgaris* broodstocks fed two different diets (mixed fish [F group, BW 1,048.14 g] or mixed crustaceans [C group, BW 998.44 g]) was analyzed using an experimental recirculating aquaculture system consisting of a tank equipped with spawning and incubation chambers. A total of 8 females (F1–4; C1–4), and 8 males (M1–M8) were selected. DI of the C group females was significantly (p < 0.05) higher (3.0 ± 0.29%) than the F group (2.16 ± 0.67%). SGR in C group was significantly higher (1.43 ± 0.12%) than the F group (1.18 ± 0.25%). Egg clusters, number of clusters, number of clusters/kg BW, and total length were more favorable in the C group than the F group. The number of clusters/kg BW of C females was 2.5 times higher than that of F females (78.1 ± 6.5 vs 31.1 ± 13.3). The total eggs number, number of eggs/cm, number of eggs/kg BW in the C group were significantly (p < 0.05) higher compared with the F group; the number of eggs/kg BW and paralarvae/kg BW were 5 times higher in the C group (115,928 ± 12,513 C vs 22,109 ± 7912 F and 114,953 ± 12,591 vs 20,729 ± 7104, respectively). Hatching rate of the C group was significantly (p < 0.05) higher compared to the F group.

## Introduction

Commercial interest in the development of octopus breeding (*Octopus vulgaris*, Cuvier 1797) has increased because cephalopod molluscs are important components of European and Italian fish markets due to high consumer demand. However, cephalopod molluscs are relatively scarce in nature due to excessive and uncontrolled harvesting, with consequent intense fluctuations in prices between periods of abundant versus inadequate supply^1,2^.

Currently, octopus is considered an innovative species for aquaculture due to a number of interesting biological traits, such as its short life cycle, high growth rate, favourable food conversion index, high fertility rate, easy adaptation to captivity, good variability in the diet, characterized by acceptance of food of low commercial value^1,3–9^. Based on these factors, there is increasing interest in Europe concerning the development of new techniques for octopus rearing, using both closed and semi-closed systems or floating cages^7^. However, several bottlenecks remain, and these have impeded the transition of technologies from pilot to full scale. One such limitation is represented by the optimization of standardized methods for controlled reproduction to obtain sufficient paralarvae production. In particular, methods of the management of paralarvae during the first part of the rearing cycle, passage from planktonic to benthic phase, need improvement due the absence of a suitable live food source to cover the paralarvae requirements^10^.

The use of a recirculating aquaculture system (RAS) could enable full control of reproduction in captivity, but various technical drawbacks associated with current methodology must be overcome to facilitate the application of these systems on an industrial scale. With respect to the common octopus, the use of hydrodynamic tanks maximizes the well-being of the paralarvae during breeding^10^. The lack of standardized reproduction techniques for use in captivity still limits aquaculture of cephalopod species (*Sepia officinalis*, *Loligo vulgaris*, *Octopus mimus*), but suitable optimized methods for embryonic development are available^11–13^.

Another critical factor that must be addressed is development of a diet for octopus broodstocks that will promote offspring production. Previous studies focused on optimizing the type of feed and its administration. Several types of foods, including bogue (*Boops boops*), and crustaceans such as the green crab (*Carcinus maenus*), yielded the best results in terms of growth, especially when using live food^5,14–16^.

Many authors are in agreement regarding the importance of a suitable diet for maximizing the reproductive performance of adult octopus^15,16^ as the same occurred in fish species^17–21^. Several studies evaluating *Octopus* sp. have highlighted the effect of maternal diet on egg quality and embryonic and paralarval development^2,22^. However, few studies have investigated the effects of diet on reproductive performance in terms of offspring production^23^ (number of eggs and paralarvae obtained).

The aim of the present study, therefore, was to evaluate the reproductive performance of common octopus (*O. vulgaris*) broodstocks fed two different diets, one based on mixed fish and the other based on mixed crustaceans. For this purpose, we employed an experimental RAS, characterized by an innovative hydrodynamic circuit, with incubation chambers, was designed with the aim of favoring spontaneous spawning of the common octopus.

## Materials and methods

### Animals

Wild sub-adult *Octopus vulgaris* were caught at the end of February using traditional “*polpara*” (a non-invasive catch system) in the Jonio Sea (Gallipoli, Puglia—Italy). Larger animals (> 700 g body weight [BW]) were selected at the catch site and then transported to the laboratory of Cesenatico, where they were categorized by weight and sex. Mean BW was recorded using an electronic scale (model WLC 20/A2, ± 0.1 g, RADWAG, Poland). Male sex was confirmed by inspecting the hectocotylus. Finally, a lot consisting of 8 females (F1–4 and C1–4, 1,094 ± 77.9 g BW) and 8 males (M1–M8, 952.6 ± 109.8 g BW) was selected.

The animals were stocked by sex in two 700-L tanks connected to a recirculating water system and allowed to acclimate for 2 days. In this system, the initial seawater temperature (15 ± 0.5 °C, salinity 35‰) and photoperiod (10.5 h light:13.5 h dark) corresponded to the octopus’ capture conditions. After acclimation, 8 couples were formed and transferred into the experimental RAS starting the reproduction trial. This study was performed in accordance with all applicable standards regarding space, as indicated in the “Guidelines for the Care and Welfare of Cephalopods in Research”^24^.

### Characteristics of the RAS

**Figure 1:**
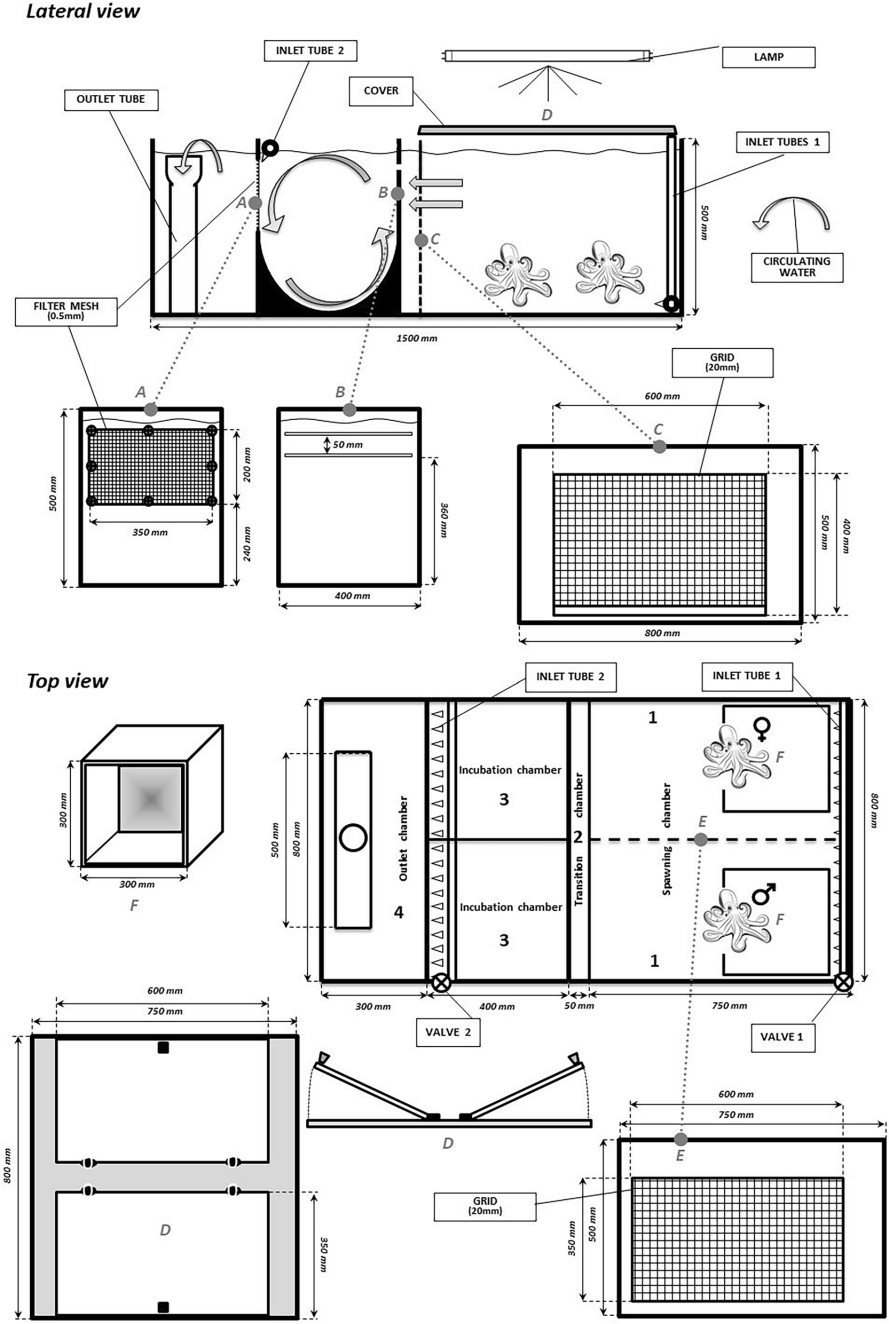
Schematic illustration of the closed recirculating system for controlled reproduction of *O. vulgaris*.

An experimental RAS, characterized by an innovative hydrodynamic circuit, with incubation chambers, was employed with the aim of favoring spontaneous spawning of the common octopus. This experimental tank (Fig. 1) was obtained by structural modification of a tank originally developed for eel reproduction designed by Mordenti et al.^25^ (Acqua&Co S.r.l. Cadelbosco di Sopra, Reggio Emilia, Italy). The RAS had a vertical configuration (rectangular plan; total volume 1.12 m^3^) and consisted of two fish-rearing tanks (0.47 m^3^/tank), a protein skimmer (0.05 m^3^), a biological filter (0.21 m^3^), and a circulating pump (max. delivery 16,000 L/h). The system was also equipped with a thermal regulation system, a UV-sterilizer lamp, an ozonizer, and an aerator. The core of the system was the reproduction tank, which consisted of five components (Fig. 1): one spawning chamber (300 L), one transition chamber (20 L), two incubation chambers (52 L), and one outlet chamber (90 L). The spawning and transition chambers were separated by a 20 mm-sized grid, raised by 40 mm from the bottom to facilitate the removing food waste. The two sectors were connected to the incubation chambers via two 5-mm lengthwise splits located on the top side of the dividing panel.

Two pipes allowed water to enter from the base of the spawning chamber (inlet tube 1) in order to guarantee water exchange and promote—once the eggs hatched—entry of the paralarvae into the incubation chambers (Fig. 1). The incubation chamber had a cylindrical base and a tube on the top (inlet tube 2), and it was provided with inlet jets that produced a circular revolving current to retain the paralarvae; an outlet mesh screen was located on the dividing panel between the incubation and outlet chambers (Fig. 1). Finally, 2 glass covers positioned on the spawning chamber functioned to maintain light conditions and trap the animals in the tank.

The spawning chamber was “naturalized” with grey-coloured walls, the presence of shells, bivalve shells, sand and stones on the bottom, and 2 dens were inserted for the octopi (Fig. 1). The spawning chamber was equipped with a removable grid to separate the broodstocks. This grid had 20 mm size of opening that was chosen to let the courtship but limiting the mating. The RAS system was evaluated for its overall functionality (water flow rate, suitability for transferring paralarvae from the reproduction chamber to the incubation chamber, suitability of the incubation camber for maintaining paralarvae).

### Experimental design

All octopi were submitted to the conditioning programm during the first 5 weeks. This time must be included in the growing phase and aimed at reaching the standard experimental conditions: temperature of 20 ± 1.0 °C (1 °C/week), photoperiod of 15.5 h light (1 h/week), salinity of 36 ± 1.0‰, pH 8.1 ± 0.1, and dissolved oxygen upper limit of 8.0 ppm. Total ammonia nitrogen (TAN), nitrite–nitrogen (NO_2_–N), and nitrate–nitrogen (NO_3_–N) were checked 3 times/week, collecting 500 cc of water for laboratory-based determination using a spectrophotometer (Hach mod-2005, Hach Co., Loveland, USA) according to APHA methods (1995).

During the reproductive period, the timing of the sequence of primary biological phases associated with reproduction of the common octopus (growing, courtship/mating, denning/spawning, hatching, senescence) was recorded. The broodstocks were subdivided into two groups (F, C) of four couples of octopi each, with each group receiving a different feeding program: the F Group (F1–M1, F2–M2, F3–M3, F4–M4) received a diet of mixed frozen (20%) and fresh (80%) fish (40% horse mackerel [*Trachurus trachurus*], 40% bogue [*Boops boops*], and 20% thinlip grey mullet [*Liza ramada*]); the C Group (C1–M5, C2–M6, C3–M7, C4–M8) received a diet of mixed frozen (20%) and fresh (80%) crustaceans (40% mantis shrimp [*Squilla mantis*], 40% common and green crabs [*Carcinus* sp*.*], and 20% caramote shrimp [*Penaeus kerathurus*]). The two diets were manually administered daily ad libitum in two meals (8 a.m.; 3 p.m.) in the reproduction chamber. When recorded, the waste was removed into the transition chamber.

When the 8 couples of octopi were formed, each was kept in the spawning chamber, initially separated by a grid, which was removed only during the early courtship stages (first contact with the ends of the arms). The grid was removed during the day and re-positioned at the evening. The sucker display from the male was assumed as the start of courtship mating phase. At the end of this phase, when the female entered the den for spawning and stopped feeding, the male was removed and transferred back to the acclimation tank. At this time, the male’s den was taken away in order to offer more space to the female. Every 4 weeks and before denning/spawning, corresponding to the end of mating and start of denning phases, usually lasting 2–3 days, the octopi from each group were individually weighed by means of an electronic scale employed in the previous activities.

### Reproductive performance

During the study, the suitability of the experimental RAS tank was tested in relation to the reproductive performance of the broodstocks and paralarvae management. The sequence of the biological phases related to *O. vulgaris* reproduction (growth, courtship/mating, denning/spawning, hatching, senescense) was evaluated in terms of days.

For all phases of the reproductive cycle, the following parameters were determined:

*Octopus*: female and male growth rate (%/day DI) as follows:

$$\left[ {\left( {PreSpawning~weight} \right.} \right. - \left. {Initial~weight} \right) \div \left. {Days~Ongrowing} \right] \div Initial~weight~ \times 100$$

and Specific Growth Rate (SGR) as follows:

$$\left[\left(\mathit{ln}PreSpawning~ weight-\mathit{ln}\left. Initial~weight\right)\div\left.Days~Ongrowing\right]\times 100\right.\right.$$

*Clusters*: total number, number/kg BW, total length and medium length; medium length was obtained from the average of 20 clusters/female by measuring.

*Eggs*: total number, number/cm of cluster, number/kg BW. Egg number and egg number/cm were calculated taking into account the average number of 5 clusters/female.

*Paralarvae*: total number, number/kg BW, hatching rate (%), survival rate at 3 dph (%) under starvation conditions. The number of paralarvae was recorded daily and calculated via volumetric estimations, counting the number of paralarvae in five 2-L samples. The paralarval survival rate at 3 dph for each female was calculated based on 3 samples of paralarvae/female just hatched. During the hatching time, (day 3, 6, 9, corresponding to start, middle and the end of the hatching) the samples were transferred into 3 incubation chambers with the same dimensional characteristics of those used in the reproduction tank and stocked at a density of 10 paralarvae/L for 72 h under starvation.

### Statistical analyses

Data concerning reproductive performances (Pre-spawning weight, DI, SGR; Clusters: total number, n./kg BW, medium and total length; Eggs: total number, n./1 cm, n./kg BW; Paralarvae: total number, n./kg BW, hatching percentage) of *O. vulgaris* broodstocks fed mixed fish (F group) and mixed crustaceans (C group) were submitted to one-way analysis of variance (ANOVA) using the model of Smith’s Statistical Programme (version 2.80, Software 2005)^26^. Differences were considered significant at P < 0.05.

All octopi were handled in accordance with the European Union regulations concerning the protection of experimental animals (Dir. 2010/63/EU).

## Results

The suitability of the experimental tank was verified by monitoring water quality parameters to ensure they remained under the limits suitable to maintain the welfare of common octopus in controlled environments. During the trial, TAN was maintained at 0.08 ± 0.2 mg/L, NO_2_–N remained at 0.07 ± 0.03 mg/L, and NO_3_–N remained at 2.1 ± 0.7 mg/L. RAS function was also evaluated at a water flow rate of 1.1 ± 0.05 L/s (0.7 ± 0.05 L/s in the reproduction chamber and 0.4 ± 0.05 L/s in the incubation chamber).

The circular water flow provided by the inlet jets kept the paralarvae in suspension in the incubation chamber and guaranteed constant cleaning of the filter mesh (i.e., self-cleaning). Neither the presence of paralarvae in the outlet chamber nor return of paralarvae from the incubation to the spawning chamber were observed.

**Figure 2:**
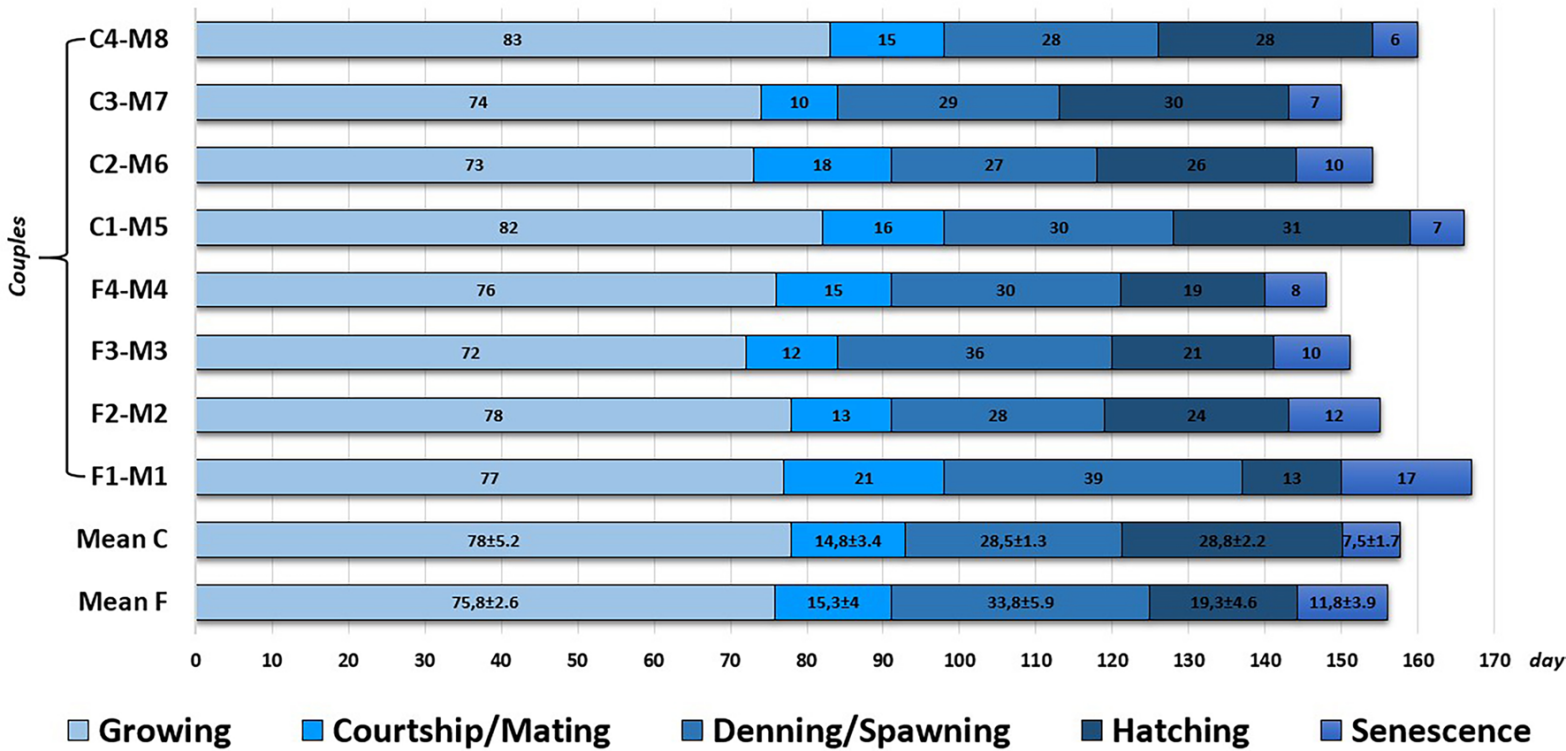
Schematic timing related the biological phases to *O. vulgaris* reproduction during the experiment. The sequence was evaluated in terms of days. The broodstocks were subdivided into two groups (Fish, F and Crustacean C) of four couples of octopi each. Mean of males (M1–M8) was coincident with relative females during the growing and courtship/mating phases.

With regard to reproductive performance in the F and C groups, the times at which the reproductive phases (growth, courtship/mating, denning/spawning, hatching, senescence) occurred are reported in Fig. 2. The reproductive cycle (from the formation of couples to the death of the females) lasted 148–167 days and was rather homogeneous among all 8 couples monitored. Also, the behaviour of all the females before spawning did not show differences in terms of loss of appetite contrary to what observed on denning. The growth phase accounted for approximately 50% of the time (49.2 ± 2.6%), whereas the courtship/mating phase accounted for approximately 10% of the time (9.2 ± 2.2%), and the denning/spawning phase represented approximately 15% of the time (15.3 ± 3.9%). In the C group, hatching lasted significantly longer (28.8 ± 2.2 days) than in the F group (19.3 ± 4.7 days). Growth rate was significantly (*p* < 0.05) higher in the C group females (DI 3.0 ± 0.29, SGR 1.43 ± 0.12) than F group females (DI 2.16 ± 0.67, SGR 1.18 ± 0.25) (Table 1). Considering average octopus growth in relation to sex, independent from diet (Table 1), the data did not show notable differences: 2.73%/day (SGR 1.36) in males and 2.40%/day (SGR 1.24) in females.

**Table 1:** Growth performances of different groups of *O. vulgaris* (Fish group, FG and Crustacean group, CG) represented in grams (g) and percentage (%) at the beginning of the study and pre-spawning.

Group	Animal	Weight			
		Initial (g)	Pre-spawning (g)	Daily increase (%)	SGR (%)
FG	F1	1,165.3	2,120.6	0.84	0.61
	F2	1,020.6	3,113.7	2.25	1.23
	F3	1,212.8	3,950.9	2.69	1.41
	F4	1,127.8	2,988.4	1.81	1.07
	M1	762.1	2,948.3	3.15	1.38
	M2	946.7	2,768.8	2.12	1.18
	M3	1,010.6	3,054.7	2.22	1.32
	M4	1,139.2	3,395.4	2.18	1.20
	Mean FG	1,048.14 ± 146.26	3,042.60 ± 519.83b	2.16 ± 0.67b	1.18 ± 0.25b
CG	C1	1,123.2	4,520.7	3.09	1.42
	C2	980.4	3,472.2	2.79	1.39
	C3	1,080.7	3,863.5	3.07	1.52
	C4	1,041.3	3,786.3	2.69	1.32
	M5	885.6	3,675.1	3.46	1.45
	M6	1,015.7	3,469.7	2.66	1.35
	M7	915.3	3,696.3	3.34	1.66
	M8	945.3	3,444.8	2.91	1.32
	Mean CG	998.44 ± 82.17	3,741.08 ± 351.51a	3.0 ± 0.29a	1.43 ± 0.12a

Different letters (a,b) on the same column show significant differences (P < 0.05) between Crustacean and Fish Group.

Regarding egg clusters, the total number of clusters, number of clusters/kg BW, and total length of clusters were more favorable in the C group than the F group. The number of clusters/kg BW was 2.5 times higher in C group females than F group females (78.1 ± 6.5 vs 31.1 ± 13.3). Similarly, the total number of eggs, number of eggs/cm, and number of eggs/kg BW observed in the C group were significantly (*p* < 0.05) higher than in the F group (Table 2). The production of eggs/kg BW and paralarvae/kg BW was 5 times higher in the C group than in the F group (115,928 ± 12,513 FG vs 22,109 ± 7912 FC and 114,953 ± 12,591 vs 20,729 ± 7104, respectively).

**Table 2:** Reproductive performances in terms of clusters, eggs and paralarvae of *O. vulgaris* females fed with fish (FG, Fish group) and crustacean (CG, Crustacean group).

Group	Female	Clusters				Eggs			Paralarvae			
		Number		Length		Number			Number		Hatching	Survival
		Total	Kg B.W.	Medium (cm)	Total (cm)	Total	1 cm	Kg B.W.	Total	Kg B.W.	%	3 DPH
FG	F1	24	11.3	8.26 ± 2.41	198	21,811 ± 25	110.2 ± 2.9	10,285	21,616 ± 105	10,193	99.1	98.2 ± 0.1
	F2	125	40.1	8.05 ± 2.11	1,006	84,123 ± 36	83.6 ± 2.4	27,017	80,124 ± 1,058	25,733	95.2	98.3 ± 0.3
	F3	142	36.0	8.11 ± 2.23	1,152	100,793 ± 48	87.5 ± 3.1	25,511	92,258 ± 1,237	23,351	91.6	98 ± 0.3
	F4	110	36.8	8.06 ± 2.17	887	76,574 ± 53	86.3 ± 1.8	25,624	70,642 ± 1,028	23,639	92.2	98.1 ± 0.2
	Mean	100.3 ± 52.5b	31.1 ± 13.3b	8.1 ± 2.2a	810.8 ± 422.6b	70,825 ± 41b	92.0 ± 2.6b	22,109 ± 7912b	66,160 ± 857b	20,729 ± 7104b	94.5 ± 3.4b	98.2 ± 0.2
CG	C1	381	84.3	7.32 ± 1.97	2,789	604,376 ± 65	216.7 ± 5.3	133,691	601,584 ± 1524	133,073	99.5	98.4 ± 0.3
	C2	241	69.4	6.64 ± 1.91	1,600	377,027 ± 32	235.6 ± 4.3	108,584	373,610 ± 987	107,600	99.1	98.5 ± 0.4
	C3	315	81.6	6.74 ± 1.87	2,123	409,236 ± 47	192.8 ± 3.2	105,924	407,113 ± 876	105,374	99.5	98.2 ± 0.3
	C4	291	76.9	7.34 ± 1.85	2,136	437,369 ± 39	204.8 ± 3.7	115,514	430,748 ± 1,147	113,765	98.5	98.3 ± 0.2
	Mean	307 ± 58.2a	78.1 ± 6.5a	7.0 ± 1.9b	2,162 ± 486.9a	457,002 ± 46a	212.5 ± 4.1a	115,928 ± 12,513a	453,263 ± 1134a	114,953 ± 12,591a	99.2 ± 0.5a	98.4 ± 0.3

Different letters (a,b) on the same column show significant differences (P < 0.05) between Crustacean and Fish Group.

The total number of paralarvae, number of paralarvae/kg BW, and hatching rate of the C group were significantly (*p* < 0.05) higher compared with the F group (453,263 ± 1134 CG vs 66,160 ± 857 FC, 114,953 ± 12,591 vs 20,729 ± 7104 and 99.2 ± 0.5 vs 94.5 ± 3.4, respectively) (Table 2). In addition, the egg cluster medium length differed significantly (*p* < 0.05) between the F (8.1 ± 2.2 cm) and C (7.0 ± 1.9 cm) groups (Table 2).

**Figure 3:**
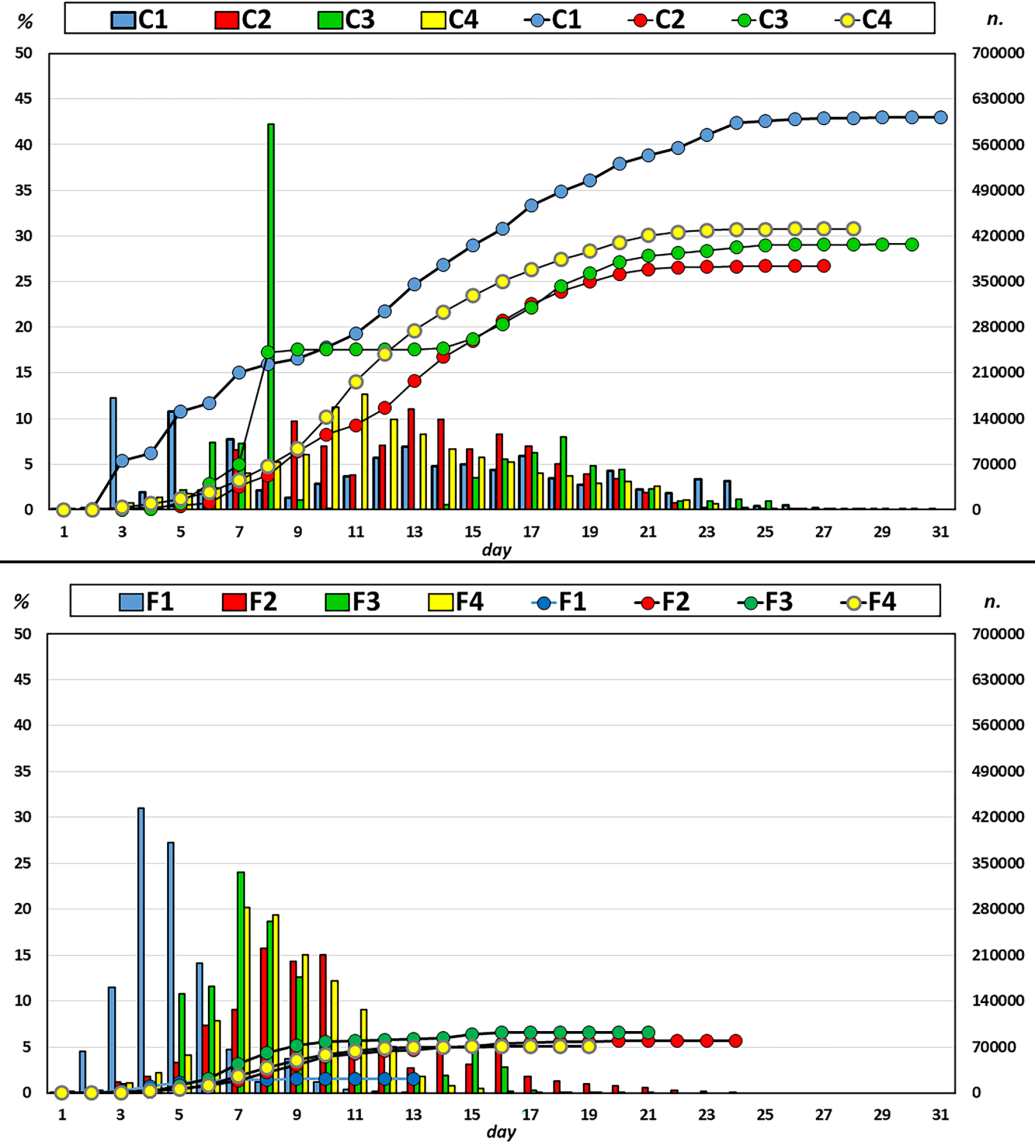
Paralarvae amount in *O. vulgaris* females fed fish (F) and crustacean (C). Squares represent percentage daily hatching (%). Circles represent cumulative number of paralarvae (n.).

With regard to egg hatching, the hatching phase lasted 13–24 days for the F group (F1 and F2 females, respectively), whereas this phase lasted 26–31 days in the C group (C2 and C1 females, respectively) (Fig. 3). For all females, an initial hatching peak was observed, with more than 50% of paralarvae hatched within a few days (in female C3, approximately 40% of the paralarvae were produced in a single day), and this rate declined over time, with the daily hatching rate declining to less than 1% in the last days (Fig. 3).

## Discussion

The experimental RAS, characterized by an innovative hydrodynamic circuit (circular current in the incubation chamber and laminar current in the remaining sectors), was designed with the aim of favoring spontaneous spawning of the common octopus. In both experimental groups, the RAS tank fully satisfied all of the requirements: the separation grid, initially placed between the male and female, prevented the territorial aggression phenomenon; the presence of the transition chamber promoted the collection of food wastes outside the spawning chamber limiting the disturb of the broodstocks; and it facilitated the natural transfer of paralarvae from the reproduction chamber to the incubation chamber up to the total hatching clusters of eggs present inside the den and cared for by the females. Throughout the study, no technical anomalies were recorded in the designed recirculating system for octopus reproduction. The tangential water flow guaranteed the complete passage of paralarvae and residual food from the spawning chamber to the incubation and transition chambers, respectively. Indeed, once egg hatching was over, no paralarvae were found within the transition or spawning chambers. In addition, the RAS allowed the females of both groups to remain undisturbed. This technique differed from a previous study in which the females were subjected to transfer to another tank after egg deposition, and such manipulation could have affected performance^27^. In our current study, every type of manipulation of the females was avoided, allowing them to care for the eggs. After removal of the males and until the senescence phase, the reproduction chamber was never opened in order to avoid stressing the females.

In the incubation chambers, the high survival rates recorded at 3 dph showed that the circular movement of the water was suitable for maintaining common octopus paralarvae under minimal stress. The survival rate in our study was higher than that of a previous study using cylindrical tanks under starvation conditions, in which the survival rate of paralarvae ranged from 37 to 70%^2^. The high survival rates recorded in our study probably resulted from the fact that in our tanks, thanks to the circular movement of water, the paralarvae did not actively swim, thus maximizing energy reserves of the yolk sac and minimizing stress. The circular water current and water flow rate were suitable to maintain octopi which, for several weeks of life, exhibit a planktonic behaviour and can easily fall into the outlet mesh screens of closed recirculating systems, with the consequent risk of mechanical shock against the walls of the tank. A previous study ascertained that rearing paralarvae under optimal conditions is the best means of maximizing growth performance and survival rate^28^. The good environmental conditions were also linked to naturalization of the reproduction chamber, where no aggressive behavior or relevant stressors (negative patterns) were observed, and all the specimens achieved reproduction. In the current study, the system tested confirmed points highlighted by Iglesias et al.^10^ regarding female breeding animals, which, when kept in captivity under suitable conditions, are able to reach maturity and produce ovarian clusters. The grey-colored tank used in the present study was more-suitable compared to the blue tanks used in previous trails^29^, in which octopus adults, without dens, succumb to autophagy and death. The designed system appeared to meet all of the technical requirements for promoting spontaneous reproduction of *Octopus vulgaris*, thus eliminating issues related to broodstocks, eggs, and paralarvae handling.

**Figure 4:**
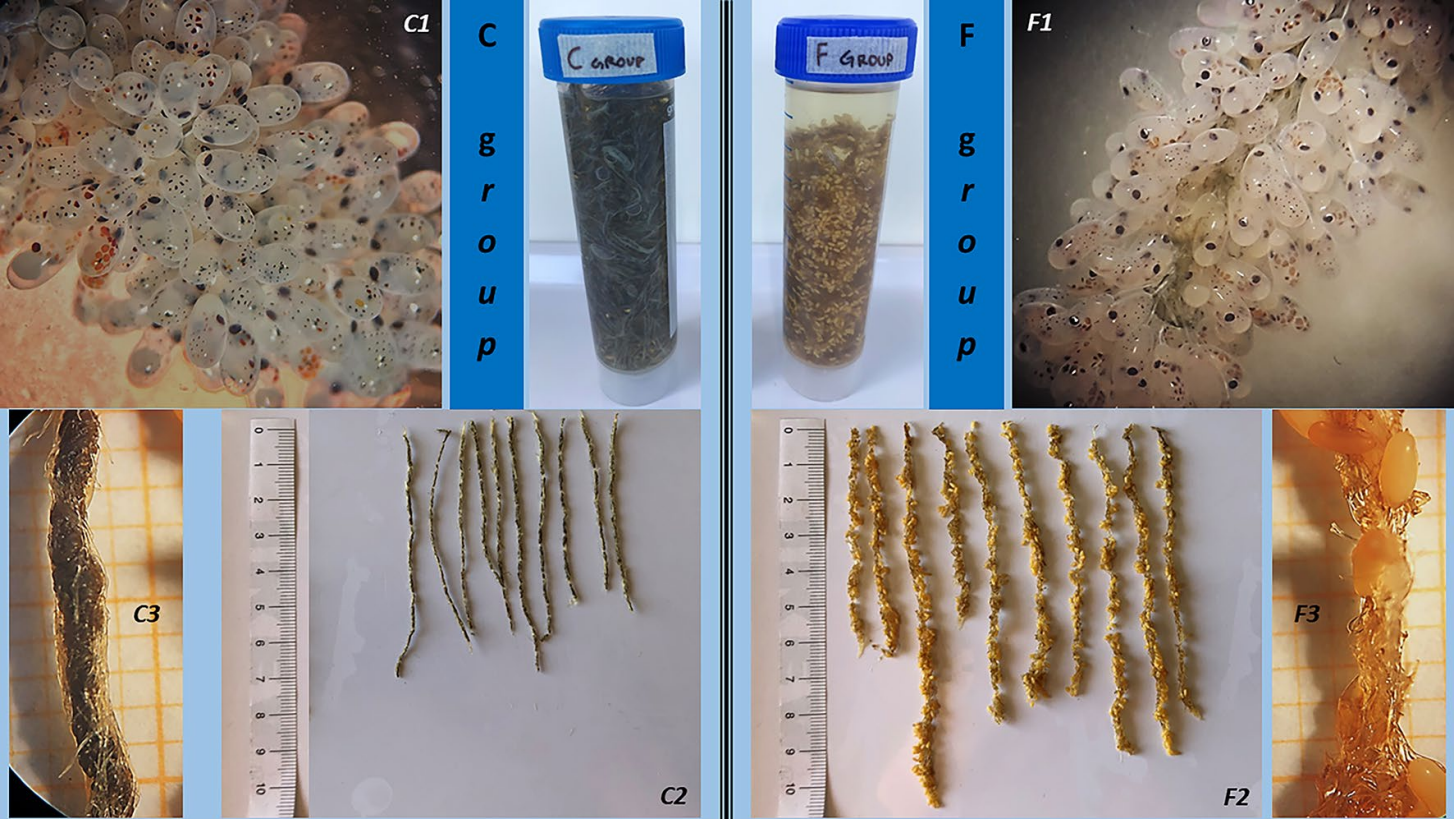
Pre/post hatching clusters of *O. vulgaris* females fed crustacean (CG, on the left of the picture) and fish (FG, on the right of the picture). (C1), (F1) show a detail of pre-spawning clusters of the groups with different density of laid eggs. (C2), (F2) show the length of the post-spawning clusters of the different groups. (C3), (F3) show sections of clusters with total (C3) and partial hatched eggs (F3).

Concerning growth, the best performance was exhibited by the octopi fed the crustacean diet (C group). This type of feed seems to better represent the diet in the wild, in which crustaceans represent 62–80% of the diet, compared to 12–30% fish^30,31^. Other studies have reported diets composed completely of crustaceans or mixed diets composed predominantly of crustaceans^15,16,32,33^. No inert diet able to completely replace fresh or frozen food and in which nutritional characteristics fluctuate in relation to the season and place of capture has yet to be developed for *O. vulgaris*^34–38^. In *Octopus* sp., the type of diet (fresh or formulated) consumed during female maturation affects various biochemical and morphologic characteristics of both embryos and hatchlings^2,23,39,40^. The results of the current study indicate that the diet composition of the broodstocks plays an essential role in determining the success of reproduction in the common octopus. As shown in Fig. 2, starting from sub-adults the feeding period (lasted 72–83 days) has been suitable to affect the spawning of the broodstocks. The present study focalizes on the relationship between diet and number of eggs and paralarvae produced and suggests that the broodstock diet strongly affects reproductive performance in terms of clusters, eggs, and paralarvae. The higher productivity of females in the C group was evidenced not only by the greater production of clusters but also by the greater density of eggs in each cluster (Fig. 4). Feeding the broodstocks a diet based on crustaceans guaranteed the highest growth and greatly improved offspring production. Conversely, the diet based on fish appeared unfavorable probably due to the unbalanced lipid content as confirmed by feces produced as filamentous, floating and fatty feces, as reported by Petza et al.^41^, not observed in the octopus group fed crustaceans.

In the group fed crustaceans, the octopus females exhibited the best reproduction performance, confirming the results of a previous study indicating that cephalopods can accumulate energy reserves in the digestive gland to be used during fasting^42^, whereas in our study no relationship was shown between fat content and egg production. According to O’Dor et al.^43^ and Lee^44^, the digestibility of lipids is low, whereas other authors reported that the capacity of octopi to metabolize lipids is very limited^45^, and their use depends on the quantity and quality of the dietary lipids^46–48^.

Concerning the octopus growth rate in relation to sex, independent from diet, our results were similar for both sexes. This result was better than those of previous studies^4,49,50^ reporting that males achieve a body weight higher than females because females direct more energy toward gonad maturation in comparison to somatic growth^3,51^. In contrast to Estefanel et al.^52^, in our study, the complete isolation of the couple and the daily presence of readily available food led the female to feed adequately up to the clogging phase, whereas we observed that males in the "frenzy of reproduction" occasionally refused food.

With regard to fertility in both relative and absolute terms, our results obtained with females fed crustaceans are in agreement with those for octopi in the wild reported by Mangold^53^, who recorded depositions of over 500,000 eggs/female. Our results were also in agreement with those reported by Iglesias et al.^14^ for captive octopus, which produced an estimated 100,000 eggs/kg BW.

As concerns denning/spawning phase, F1 and F3 females showed that a longer phase of eggs spawning is not in relationship with a number of eggs spawned. Evidently, once entered the den, the start of spawning and number of clusters daily produced varies from female to female.

The hatching rate was very high in both groups in the present study, exceeding 90%, and the rates were in line with those reported by Iglesias and Fuentes^26^, who reported values above 80% in captive octopi. In this regard, it is important to emphasize how the newly designed RAS enabled the collection and separation of paralarvae of the same age, a fundamentally important capability that emerged from a study of proper management techniques in a larval feeding program^54^. In general, the different types of diet adopted did not affect the behavioral and reproductive timing of the couples tested. The longer duration of the hatching phase in the C females was due to the greater egg production, which necessitated a longer period for laying and hatching. Knowledge of aspects such as the timing of mating and laying eggs is certainly useful for optimizing reproduction in a controlled environment and with respect to closing the production cycle.

Identifying technical and hydrodynamic solutions for rearing these cephalopods in captivity is very important, not only to ensure animal welfare but also for reproductive purposes. The reproduction system tested in this study exhibited all the necessary requirements to induce mating and allow spontaneous captive reproduction of *O. vulgaris*. The new RAS consisting of a reproduction tank with space for each single couple enabled determination of the daily hatching rate of paralarvae as well as observation of the female behaviour in the denning phase. The size of the transition chamber was suitable to remove uneaten feed. However, it would be plausible to increase its size so as to use it as feeding chamber also, further reducing the disturbing action. The current study demonstrated that this system enables separation of the daily hatchlings, with the consequent advantage of easy planning of paralarvae weaning because of the possibility of starting from more homogenous paralarvae lots. Furthermore, the hydrodynamic conditions of the plant did not hinder the reproductive activities of the specimens and favored the spontaneous transfer of paralarvae to the incubation chambers immediately after hatching, thus avoiding the need for any type of manipulation and preventing mechanical stress phenomena. In addition, the circular water flow of the system facilitated maintaining the paralarval forms in suspension. With respect to the diet adopted for broodstocks of the common octopus, a diet based on crustaceans not only favored better growth rates but above all enhanced reproductive performance in terms of the quantity of eggs produced and consequently of paralarvae obtained.

## Supplementary information

Supplementary Video.

## Abbreviations

BW: Body weight
DI: Daily increase
SGR: Specific growth rate
