# Author Correction: Evaluation of reproductive performances of the common octopus (*Octopus vulgaris*) reared in water recirculation systems and fed different diets

**DOI:** 10.1038/s41598-022-16756-5

**Published:** 2022-08-01

**Authors:** Antonio Casalini, Alessandra Roncarati, Pietro Emmanuele, Niccolò Guercilena, Alessio Bonaldo, Luca Parma, Oliviero Mordenti

**Affiliations:** 1 Department of Veterinary Medical Sciences – DIMEVET, University of Bologna, Via Tolara di Sopra 50, 40064 Ozzano dell’Emilia, BO Italy; 2 School of Biosciences and Veterinary Medicine, University of Camerino, Viale Circonvallazione 93/95, 62024 Matelica, MC Italy

Correction to: *Scientific Reports* 10.1038/s41598-020-72151-y, published online 17 September 2020

This Article contained an error in that it included a number for an ethical approval for another study. Under relevant national and European Union regulations, the research described in this paper does not require authorisation from an ethics committee, which was confirmed by the Animal Welfare Committee of the University of Bologna.

As a result, in the Materials and methods section,

"All octopi were handled in accordance with the European Union regulations concerning the protection of experimental animals (Dir. 2010/63/EU). Approval for this study was obtained from the Ethics Committee of Bologna University (prot. 19/6912)."

now reads:

"All octopi were handled in accordance with the European Union regulations concerning the protection of experimental animals (Dir. 2010/63/EU)."

The original Article has been corrected.

